# Leader Psychological Need Satisfaction Trickles Down: The Role of Leader-Member Exchange

**DOI:** 10.3389/fpsyg.2022.799921

**Published:** 2022-04-25

**Authors:** Anouk Decuypere, Robin Bauwens, Mieke Audenaert

**Affiliations:** ^1^DigiTax Research Center, Research Group of Business & Law, Faculty of Law, Antwerp University, Antwerp, Belgium; ^2^Department of Human Resource Studies, Tilburg School of Social and Behavioral Sciences, Tilburg University, Tilburg, Netherlands; ^3^Department of Marketing, Innovation and Organization, Research Group Human Resource Management and Organizational Behavior, Faculty of Economics and Business Administration, Ghent University, Ghent, Belgium

**Keywords:** trickle-down, multilevel, psychological need satisfaction, leader-member exchange, well-being, leadership, leader wellbeing, employee wellbeing

## Abstract

This article addresses the impact of leader psychological need satisfaction on employees. We draw on the self-determination theory (SDT) and leader-member exchange (LMX) theory to investigate if and how leader psychological need satisfaction trickles down to employee psychological need satisfaction. Adopting a multi-actor, multilevel design, results from 1036 leader–employee dyads indicate that employee-rated LMX mediates the trickle-down effect of leader psychological need satisfaction. Additional analyses of leader psychological needs show that leader competence is the main psychological need that underlying this relationship. We also found an unexpected negative association between leader autonomy need satisfaction and employee competence need satisfaction. Overall, this study shows the importance of both (1) leaders’ psychological need satisfaction and (2) employee perceptions of the relationship quality for employee psychological need satisfaction.

## Introduction

Psychological need satisfaction is no novel concept to leadership researchers. With its origins in the self-determination theory (SDT; Deci and Ryan, 2008), psychological need satisfaction advances that fostering autonomy, competence, and relatedness will lead to autonomous motivation at work. This is true for employees, but also for leaders themselves: when leaders’ psychological needs are satisfied, those leaders have more energy to perform behaviors that are in accordance with positive leadership styles (Trépanier et al., 2012; Paas et al., 2020). However, despite psychological need satisfaction’s prominent position in leadership research, for example, in studies on the transformational leadership (Breevaart et al., 2014a), empowering leadership (Chen et al., 2021) or inclusive leadership (Chiniara and Bentein, 2016), a few shortcomings can be noted. First, psychological need satisfaction has mostly been examined as a consequence of leadership. Accordingly, recent research indicates it is also important to consider psychological need satisfaction as an antecedent of positive leadership styles, as to better understand why leaders engage into behaviors conductive of such styles (Paas et al., 2020). Second, prior work has mostly adopted a single level approach to psychological need satisfaction, overlooking how psychological need satisfaction is embedded in complex relations between leaders and followers (Kuvaas and Buch, 2019; Xie et al., 2020) that cross multiple levels of analysis (Schreurs et al., 2014; Batistič et al., 2017).

Addressing this research gap, the aim of this study is to examine how leaders’ psychological need satisfaction trickles down to the psychological need satisfaction of their employees. Trickling down’ refers to interaction patterns or perceptions that cascade to different levels in the organization (Ambrose et al., 2013; Jeuken, 2016; Jiang and Lin, 2021; Wu et al., 2021; Zhong et al., 2021). Indeed, psychological need satisfaction might be an important element in determining how leaders act and subsequently on how employees come to evaluate and react to leaders (Decuypere and Schaufeli, 2020, 2021). In particular, we advance that this trickling down takes place through interpersonal exchange processes between leaders and employees, as exemplified by leader-member exchange (LMX). LMX refers to the fact that leaders develop differentiated relationships with their employees (Graen and Uhl-Bien, 1995), where high-quality relationships are characterized by exchanges based on mutual trust, respect, liking, and influence (Liden and Maslyn, 1998) and lead to positive outcomes for both parties (Graen and Uhl-Bien, 1995; Dulebohn et al., 2012; Xie et al., 2020). Since LMX is considered as need-driven (Dulebohn et al., 2012) and need-satisfying process (Kuvaas and Buch, 2019) that connects multiple levels of analysis (Liao et al., 2019), we propose that LMX constitutes a mechanism through which leader psychological need satisfaction trickles down to employees, from a dyadic viewpoint.

Overall, this paper contributes to the literature in several ways. First, we add to theorizing on psychological need satisfaction by strengthening research that views psychological need satisfaction as a consequence of the employee–leader relationship. We also explore its role as an antecedent and expand SDT by incorporating a trickle-down perspective. Second, we focus on the dyadic perspective on LMX. This perspective is less prevalent (Krasikova and LeBreton, 2012; Schyns and Day, 2010), but important since meta-analyses show that leader and employee ratings of LMX are only moderately related (Sin et al., 2009; Schyns and Day, 2010). Third, examining LMX as one of the mechanisms underlying the trickle-down effect of psychological need satisfaction from leaders to employees is topical in light of recent calls for the further integration of LMX and SDT (Andersen et al., 2020). Finally, from a practical point of view, this study highlights the necessity of focusing on leader self-determination as a way to enhance leaders’ sense of autonomy, competence, and relatedness, while benefitting employees’ psychological experiences as well.

### The Relationship Between Leaders’ and Employees’ Psychological Need Satisfaction

To argue the trickle-down of leaders’ to employees’ psychological need satisfaction, we build on SDT, because it “currently provides the best-validated and most parsimonious set of fundamentally satisfying psychosocial experiences, by making a strong empirical case for the existence of three basic psychological needs, the fulfillment of which is essential for human wellness” (Martela and Sheldon, 2019, p. 459). Furthermore, by adopting SDT as a theoretical lens, we follow but also extend contemporary debates on linking leadership behavior to follower outcomes (Chiniara and Bentein, 2016; Ju et al., 2019; Decuypere and Schaufeli, 2020; Chen et al., 2021; Stremersch et al., 2021). According to SDT, leaders that are intrinsically motivated are more inclined to invest in the well-being and psychological growth of their employees; they will invest more in the (exchange) relationships with their employees when their own basic psychological needs are met (Deci and Ryan, 2008). Accordingly, we propose that the satisfaction of each of leaders’ three basic needs (autonomy, competence, and relatedness) will motivate those leaders to behave more generously in exchange relationships with their employees (i.e., LMX), providing those employees with more resources that ultimately also satisfy their own needs.

#### The Need for Autonomy

Autonomy need satisfaction is related to “experiencing choice and feeling like the initiator of one’s own actions” (Baard et al., 2004, p. 2046) or “experiencing a sense of volition and psychological freedom” at work (Van den Broeck et al., 2010, p. 981; Van den Broeck et al., 2021). When leaders feel depleted in autonomy need satisfaction, they might seek to restore their resources (Deci and Ryan, 2008) by being more defensive and reluctant to give up control (Hodgins et al., 2006; Van Quaquebeke and Felps, 2016; Lund, 2020). Conversely, when leaders’ need for autonomy is satisfied, they will feel less threatened by the idea of providing employees with similar autonomy (Hodgins et al., 2006), translating into more autonomy-supportive behavior (Van Quaquebeke and Felps, 2016) that benefits employees’ autonomy need satisfaction.

#### The Need for Competence

Competence need satisfaction is related to “succeeding at optimally challenging tasks and attaining desirable outcomes” (Baard et al., 2004, p. 2046) or put more succinctly: “feeling effective” (Van den Broeck et al., 2010, p. 981; Van den Broeck et al., 2021). Leaders who feel insecure are depleted in competence need satisfaction and tend to feel less secure about their leadership capabilities (Paas et al., 2020). Accordingly, such leaders may ask less genuine questions in conversations with employees (Van Quaquebeke and Felps, 2016) or might be more distracted because they focus on their own insecurities (Kahn, 1990). This may lead to poorer decision making, but also to less concern for the employees’ perspective, which is crucial for relationship building (Meinecke and Kauffeld, 2019). In addition, a lack of self-perceived leader competence is related to leader aggression (Fast and Chen, 2006) and stress. In turn, this has demonstrable effects on employee stress and affective well-being (Skakon et al., 2010), at the detriment of employees’ competence need satisfaction. Conversely, leaders’ that perceive their need for competence satisfied, will feel secure about their capabilities and will ask more genuine questions to their employees (Van Quaquebeke and Felps, 2016). In turn, this provides employees with the guidance, feedback (Gagné and Deci, 2005), and psychological safety (Mao et al., 2019) that is necessary to build confidence and capabilities. In a similar vein, studies have shown that leadership from competent leaders is positively related to employee’s psychological need satisfaction (Breevaart et al., 2014a; Reb et al., 2014; Van Dierendonck et al., 2014).

#### The Need for Relatedness

Relatedness need satisfaction means “establishing a sense of mutual respect and reliance with others” (Baard et al., 2004, p. 2046) or simply as feeling a sense of connection and belonging on the work floor (Van den Broeck et al., 2010, 2021). Leader relatedness need satisfaction impacts joviality and openness of their communication with employees. When relatedness need satisfaction is depleted, leaders can experience social insecurity (Van Quaquebeke and Felps, 2016) that diminishes relatedness need satisfaction. On the contrary, leader relatedness need satisfaction may translate into social behaviors toward employees that positively influence the employees’ relatedness need satisfaction. When leaders feel a sense of connection with their employees, employees are likely to feel the same sense of belonging as well (Uhl-Bien, 2006; Zheng et al., 2020).

In sum, when leaders’ needs for autonomy, competence, and relatedness are satisfied, leaders may be more inclined to grant employees autonomy and support, boost the confidence levels of employees, and ask them genuine questions that foster employee relationships (Van Quaquebeke and Felps, 2016). In turn, both leaders and employees enjoy a level of decision-making freedom, competence, fulfillment, and optimal performance:

*Hypothesis 1: Leader psychological need satisfaction is positively and directly related to employee psychological need satisfaction.*

### The Mediating Role of Leader-Member Exchange

#### Leader Psychological Need Satisfaction and Leader-Member Exchange

First, when leaders’ basic psychological needs are satisfied, they are more likely to treat employees respectfully (Scandura, 1999; Masterson et al., 2000) and engage in genuine dialog (Van Quaquebeke and Felps, 2016), which benefits high-quality relationships with employees, from both the perspective of the leader and the employee. Second, leader psychological need satisfaction is related to less aggression, defensiveness, and social insecurity (Fast and Chen, 2006; Hodgins et al., 2006; Van Quaquebeke and Felps, 2016), which will enhance perceived fairness of employee treatment, and therefore, interpersonal justice. Perceptions of fairness and justice are associated with higher LMX-quality as well (Graen and Scandura, 1987; Scandura, 1999; Sparr and Sonnentag, 2008; Reb et al., 2018).

Taken together, we propose:

*Hypothesis 2a: Leader psychological need satisfaction is positively related to leader-rated LMX.*

*Hypothesis 2b: Leader psychological need satisfaction is positively related to employee-rated LMX.*

#### Leader-Member Exchange and Employee Psychological Need Satisfaction

We also propose that high-quality LMX from both the leader’s and the employee’s perspective influences employee psychological need satisfaction. On the one hand, high-quality *leader-perceived LMX* may motivate leaders to provide employees with more resources, like autonomy and support (Graen and Uhl-Bien, 1995). Furthermore, a high LMX relationship will satisfy the employees’ need for relatedness through friendship and a sense of belonging shared with the leader (Van den Broeck et al., 2010). On the other hand, high-quality *employee-perceived LMX* may also result in enhanced employee psychological need satisfaction, and this through similar mechanisms. High-quality LMX is characterized by high levels of trust and support (Liden and Maslyn, 1998; Andersen et al., 2020), which provide the employees with the appropriate relational environment to ask for more autonomy or support when needed. Trust may serve as a foundation to have difficult conversations, like giving and receiving feedback (Peterson and Jackson Behfar, 2003). In addition, this feedback is also more appreciated, since high-quality LMX is associated with more respect for each other’s contributions (Graen and Uhl-Bien, 1995), adding to employees’ effectiveness and competence need satisfaction (Van den Broeck et al., 2010). Likewise, high employee-perceived LMX will lead to a higher relatedness need satisfaction, since LMX is a relational process that entails the development of professional relationships and which has a sense of belonging at its core (Van den Broeck et al., 2010).

Therefore, we hypothesize:

*Hypothesis 3a: Leader-rated LMX is positively related to employee psychological need satisfaction.*

*Hypothesis 3b: Employee-rated LMX is positively related to employee psychological need satisfaction.*

Combining the reasoning outlined above (H2a, H2b, H3a, and H3b) and the dyadic perspective in LMX theory (Krasikova and LeBreton, 2012; Gooty and Yammarino, 2011; Gooty et al., 2012; Tse et al., 2018), we further hypothesize that the LMX-perceptions of both leaders and employees are relevant in the trickle-down relationship of leader psychological need satisfaction. This notion is also supported by research on respectful inquiry, which proposes that psychological need satisfaction trickles down from leaders to followers through positive, open, and respectful communication (Van Quaquebeke and Felps, 2016), forming the basis of high-quality relationships (Uhl-Bien, 2006).

**FIGURE 1:**
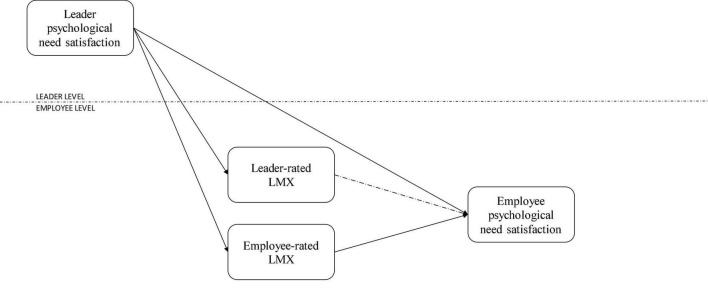
Research model.

This leads us to the last hypotheses (see Figure 1 for the research model):

*Hypothesis 4a: Leader-rated LMX mediates the trickle-down effect of leader psychological need satisfaction on employee psychological need satisfaction.*

*Hypothesis 4b: Employee-rated LMX mediates the trickle-down effect of leader psychological need satisfaction on employee psychological need satisfaction.*

## Materials and Methods

### Sample and Procedure

Data collection took place in elderly care homes in Flanders (Belgium). We focus on nurses given that the broader healthcare sector presents a challenging context where employees and their leaders are challenged by stressful circumstances, high work pressures, demanding patients and shift work (Smith, 2014; Tahghighi et al., 2017; Chênevert et al., 2019). The current pandemic has only exacerbated such circumstances, necessitating additional research to develop appropriate leader responses (Bauwens et al., 2021). Health policy reports (e.g., Rafferty et al., 2019) show that Belgium nurses face comparable professional challenges and remuneration compared to their European counterparts. In addition, Belgium nurses have often featured in prior studies on leadership (e.g., Van Bogaert et al., 2015; Van Hecke et al., 2019; Audenaert et al., 2020).

In the autumn of 2017, we recruited a sample of nurse-head nurse dyads within elderly care homes. Prior to the data collection, the directors of the nursing homes were briefed about the purpose and nature of the research and invited to participate. From the 392 elderly care homes that were contacted, 108 participated in the study, which results in a response rate of 28%. We used a paper-and-pencil questionnaire with sealed, anonymous envelopes. We received responses from 283 head nurses and 1045 nurses. After a matching procedure through unique and anonymized codes, 1,036 nurse-head nurse dyads – clustered within elderly care wards – could be retained. Informed consent was obtained from the director and each participant. Nurses were predominantly female (91.70%). On average, they were 38.79 years old (*SD* = 11.35) and had 14.96 years of experience (*SD* = 9.20). Most head nurses were female (80.4%), 45.38 years old (*SD* = 9.69), had 11.50 years of experience in their role (*SD* = 8.03) and supervised on average 19.24 nurses in his or her ward (*SD* = 8.99).

### Measures

We used scales with established psychometric properties and adopted a seven-point Likert scale for each questionnaire (1 = totally disagree, 7 = totally agree). Leaders rated their own psychological needs satisfaction and LMX with regards to each individual employee.

Head nurses were asked to rate a maximum of four nurses in order to make the data-collection feasible. As a consequence, this may have led to selection effects, whereby head nurses could have selected nurses they had a more favorable relationship with or with whom they have more contact. In order to counteract this potential bias, we asked them to report nurses alphabetically.

Employees rated LMX with their leader in addition to their own psychological need satisfaction. Items were administered in Dutch, using valid translations from previous studies.

#### Psychological Need Satisfaction

Psychological need satisfaction was assessed with the Dutch version of the Work-Related Basic Need Satisfaction Scale (Van den Broeck et al., 2010). This scale distinguishes between autonomy (e.g., “I feel free to do my job the way I think it could best be done”), competence (e.g., “I feel competent at my job”), and relatedness (e.g., “I don’t really feel connected with other people at my job,” reversed). Both leaders’ and employees’ psychological need satisfaction demonstrated good internal reliabilities with alpha 0.87 and 0.84, respectively.

#### Leader-Member Exchange

Leader-member exchange was measured with the eight-item scale by Bauer and Green (1996)^1^. We obtained Dutch items from Audenaert et al. (2019). One item loaded insufficiently on its factor (λ < 0.40) and was removed from the leader-rated LMX scale. For comparability reasons, we also removed this item from the employee-rated LMX scale (“[I/My leader], would bail [me/this employee] out, even if this is at [my/his/her] expense”). Leader-rated LMX and employee-rated LMX had respective alphas of 0.89 and 0.90.

#### Control Variables

We controlled for gender, tenure and span of control (SPOC). First, gender has been associated with possible differences in psychological need satisfaction, more specifically relatedness (Baard et al., 2004). Second, leader tenure is likely associated with leader levels of autonomy and competence. The (relationship) tenure has also been shown to be associated with LMX quality (Maslyn and Uhl-Bien, 2001; Sin et al., 2009). Third, we also controlled for SPOC, since the organizational context influences how dyads function (Graen and Scandura, 1987; Schyns and Day, 2010). Furthermore, a large SPOC might complicate the development of high-quality relationships with all employees (Cogliser et al., 2009; Schyns and Day, 2010).

### Analytical Strategy

Our model is designed as two 2–1–1 mediations, with individual nurses nested in wards supervised by a head nurse. The intraclass correlations (ICCs) demonstrated that 21.50% of the variance in employee-rated LMX, 35.55% of the variance in leader-rated LMX, and 4.26% of the variance in employee psychological need satisfaction were situated at ward or head nurse-level, warranting the use of multilevel techniques. First, we tested the convergent and discriminant validity of the measurement model with multilevel confirmatory factor analysis (MCFA). Following Kline (2015), we respected cut-off values of ≥0.90 for the Comparative Fit Index (CFI) and Tucker–Lewis Index (TLI), ≤0.08 for the Root Mean Square Error of Approximation (RMSEA) and Standardized Root Mean Square Residual (SRMR). To combat potential negative effects of negatively worded items on the covariance structure, we used item parceling for positively and negatively worded (reverse) items from the psychological need satisfaction scale (Zhang et al., 2016). Second, we examined our hypotheses with hierarchical regression analyses. For each model, we calculated the pseudo explained variance (pseudo *r*^2^) for each level of analysis (Bliese and Bliese, 2016), as well as the total explained variance (total *r*^2^) using the following formulae: total *r*^2^ = pseudo *r*^2^ level 1 × [1 − ICC(1)] + pseudo *r*^2^ level 2 × ICC(1). Finally, we assessed the multilevel mediation through Monte Carlo simulations (Preacher and Selig, 2012). Analyses were performed in R with the packages *lavaan* (Rosseel, 2012) and *nlme* (Pinheiro et al., 2017).

## Results

### Measurement Model

The MCFA models and fit indices can be consulted in Table 1 below. The results showed that the hypothesized four-factor model (i.e., leader psychological need satisfaction, leader-rated LMX, employee-rated LMX, and employee psychological need satisfaction) had a good fit to the data, with acceptable fit indices (χ^2^ [578] = 1489.48, *CFI* = 0.92, *TLI* = 0.93, RMSEA = 0.04, SRMR = 0.08). All items loaded on their respective factors (λ > 0.40; range: 0.44–0.93), excluding two items that were previously removed (see “LMX” under “measures”). Since a one-factor model (Δχ^2^ = 3765.34, Δ*df* = 364, *p* < 0.001) and a common factor model (Δχ^2^ = 1007.61, Δ*df* = 45, *p* < 0.001) fitted the data significantly worse, considerable common source bias could be ruled out. Furthermore, an eight-factor model (i.e., psychological need satisfaction scales as separate dimensions) only fitted the data marginally better (Δχ^2^ = 8.67, Δ*df* = 4, *p* < 0.10). Therefore, we chose to retain the hypothesized model for the main analyses.

**TABLE 1:** Models and fit indices.

	χ^2^ (df)	Δχ^2^ (df)	CFI	TLI	RMSEA	SRMR
Four-factor model (PNS total)	1489.49 (578)		0.92	0.91	0.04	0.08
Eight-factor model (PNS subcomponents)	1480.82 (574)	8.67 (4)	0.92	0.91	0.04	0.09
One-factor model (CSB)	5254.82 (942)	3765.34 (364)***	0.55	0.53	0.08	0.09
Common factor model (CSB)	2497.09 (623)	1007.61 (45)***	0.82	0.80	0.06	0.06

CFI, comparative fit index; TLI, Tucker–Lewis index; RMSEA, root mean square error of approximation; PNS, psychological need satisfaction; SRMR, standardized root mean square residual; CSB, common source bias. ***p < 0.001, Δχ^2^ was based on the comparison withthe hypothesized four-factor model.

### Descriptive Statistics and Correlations

Table 2 reports the descriptive statistics and correlations. Leaders’ gender, as well as employees’ gender and tenure were unrelated to the focal constructs. Leader tenure was positively related to the leader-rated LMX (*r* = 0.11, *p* < 0.01) and psychological need satisfaction (*r* = 0.19, *p* < 0.01). Leaders’ SPOC was negatively associated with leader-rated need satisfaction (*r* = −0.09, *p* < 0.01). Leader-rated LMX was positively associated to leader psychological need satisfaction (*r* = 0.24, *p* < 0.01). Employee-rated LMX showed positive correlations with employee psychological need satisfaction (*r* = 0.46, *p* < 0.01). Finally, the correlation between leader-rated LMX and employee-rated LMX was small, but significant (*r* = 0.17, *p* < 0.001).

**TABLE 2:** Descriptive statistics and correlations.

		Mean	SD	1	2	3	4	5	6	7	8
	**Leader level**										
1	Leader gender	0.80	0.40								
2	Leader tenure	11.27	8.04	–0.10							
3	Leader SPOC	19.24	8.89	–0.01	0.06						
4	Leader-rated LMX	5.64	0.62	0.01	0.13*	0.09	(α = *0.89*)				
5	Leader need satisfaction	5.59	0.67	0.02	0.19**	−0.10	0.33**	(α = *0.87*)			
6	*Leader autonomy*	5.31	0.86	0.00	0.10	−0.07	0.24**	0.87**	(α = *0.82*)		
7	*Leader competence*	5.74	0.76	–0.04	0.23**	−0.03	0.27**	0.76**	0.51**	(α = *0.65*)	
8	*Leader relatedness*	5.74	0.82	–0.08	0.15*	−0.14*	0.30**	0.81**	0.59**	0.37**	(α = *0.79*)
	**Employee level**										
1	Employee gender	0.92	0.28								
2	Employee tenure	14.96	9.20	0.05							
3	Employee-rated LMX	5.52	0.91	–0.03	0.04	(α = *0.90*)					
4	Employee need satisfaction	5.45	0.64	0.01	0.00		(α = *0.84*)				
5	*Employee autonomy*	4.97	0.95	0.03	0.02	0.42**	0.80**	(α = *0.79*)			
6	*Employee competence*	5.83	0.68	–0.03	–0.04	0.27**	0.68**	0.38**	(α = *0.78*)		
7	*Employee relatedness*	5.31	0.92	0.00	0.00	0.32**	0.76**	0.36**	0.29**	(α = *0.78*)	

Gender was coded as 1 = female, 0 = male. *p < 0.05; **p < 0.01; *** p < 0.001; N employees = 1045; N leaders = 283.

### Hypothesis Testing

Table 3 presents the results of the hierarchical regression analyses. All coefficients are unstandardized. Effects of the control variables were largely absent, although leader tenure was associated with higher leader-rated LMX (*b* = 0.01, *p* < 0.05) and employee-rated LMX (*b* = 0.01, *p* < 0.05). In addition, a higher SPOC corresponded to lower employee-rated LMX (*b* = −0.01, *p* < 0.05). Congruent with Hypothesis 1, leader psychological need satisfaction was directly related to employee psychological need satisfaction (*b* = 0.11, *p* < 0.01). Leader psychological need satisfaction also predicted both leader-rated LMX (*b* = 0.30, *p* < 0.001) and employee-rated LMX (*b* = 0.19, *p* < 0.001), conforming to Hypothesis 2a and Hypothesis 2b. However, while employee-rated LMX was associated with the employee need satisfaction (*b* = 0.34, *p* < 0.001), this was not the case for leader-rated LMX (*b* = −0.03, *p* > 0.05). Therefore, we can confirm Hypothesis 3b, but not Hypothesis 3a.

**TABLE 3:** Hierarchical regression results for the final model.

	Leader-rated LMX		Employee-rated LMX		Employee’s psychological need satisfaction	
	*B*	*SE*	*b*	*SE*	*b*	*SE*
Intercept	3.63***	0.41	4.57***	0.44	2.99***	0.26
Leader gender	–0.00	0.11	0.06	0.12	–0.02	0.06
Employee gender	0.07	0.12	–0.02	0.13	0.09	0.08
Leader tenure	0.01*	0.01	0.01*	0.00	0.00	0.00
Employee tenure	0.00	0.00	0.00	0.01	–0.00	0.00
SPOC	0.00	0.00	−0.01*	0.00	–0.00	0.00
Leader psychological need satisfaction	0.30***	0.07	0.19***	0.07	0.10**	0.04
Leader-rated LMX					–0.03	0.03
Employee-rated LMX					0.35***	0.02
Pseudo *r*^2^ lv1	0.11		0.01		0.24	
Pseudo *r*^2^ lv2	0.29		0.09		0.03	
Total *r*^2^	0.18		0.02		0.20	

*p < 0.05; **p < 0.01; ***p < 0.001. Total r^2^ = pseudo r^2^ level 1 × [1 − ICC(1)] + pseudo r^2^ level 2 × ICC(1), therefore only the total r^2^ (not the pseudo r^2^s) can be compared across models.

**FIGURE 2:**
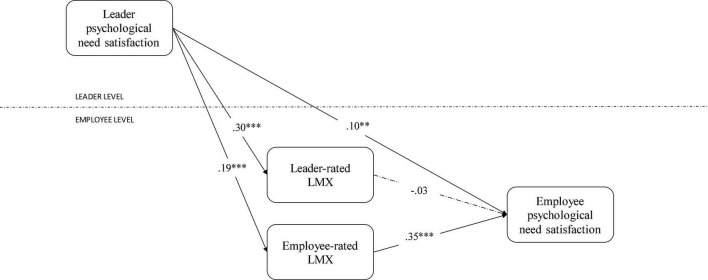
Hierarchical regressions. **p < 0.01; ***p < 0.001.

Subsequently, we assessed the mediation hypotheses. Since leader psychological need satisfaction was (a) related to employee need satisfaction, as well to (b) employee-rated LMX and (c) the latter variables were also related to each other, we assessed the indirect effect through Monte Carlo mediation. As indicated in Table 4, the average indirect effect across groups was 0.07 (*p* < 0.02), 95% *CI* [0.02; 0.12], and the total effect was 0.17 (*p* < 0.001), 95% *CI* [0.08; 0.25], providing support for Hypothesis 4b. Since leader psychological need satisfaction (a) related to employee need satisfaction, as well to (b) leader-rated LMX, but (c) the latter variables showed no significant relations, we could reject Hypothesis 4a. See Figure 2 for a visualization of research results.

**TABLE 4:** Monte Carlo mediation for leader psychological need satisfaction.

Effect	*b*	CI lower	CI upper
Direct	0.10*	0.02	0.18
Indirect	0.07**	0.02	0.12
Total	0.17***	0.08	0.25

*p < 0.05; **p < 0.01; ***p < 0.001.

### Additional Analyses

**FIGURE 3:**
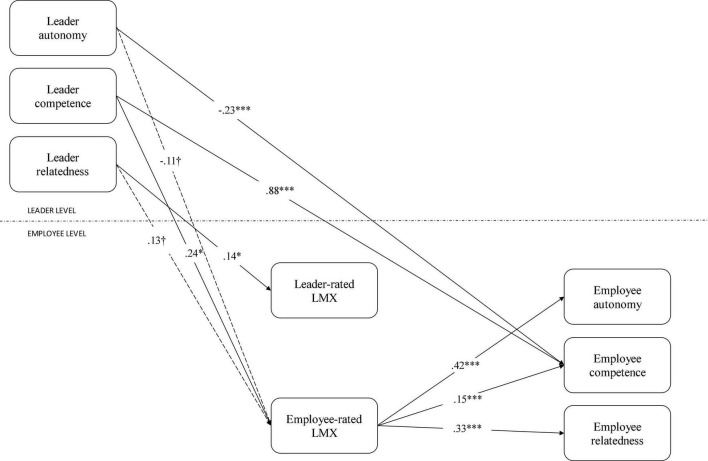
Hierarchical regressions per psychological need. ^†^*p* < 0.10; *p < 0.05; ***p < 0.001.

Since a model with differential psychological need satisfaction (i.e., autonomy, competence, and relatedness) also presented good fit indices, we calculated the regression results for models where both leader and employee psychological need satisfaction were presented by their separate dimensions. The ICCs for the separated psychological needs demonstrated that 8.98% of the variance in autonomy need satisfaction, 1.7% of the variance in competence need satisfaction and 5.97% of the variance in relatedness need satisfaction was situated at the ward or head nurse-level. The full results of the hierarchical regressions can be consulted in Table 5. The full results for the Monte Carlo mediation effects can be found in Table 6. For visualization, see Figure 3. With regards to the control variables, we found that employee autonomy was lower in the presence of female leaders (*b* = −0.16, *p* < 0.05), but also higher for female employees (*b* = 0.23, *p* < 0.05). Furthermore, a higher SPOC corresponded to lower employee relatedness (*b* = −0.01, *p* < 0.01).

**TABLE 5:** Hierarchical regressions per psychological need.

	Leader-rated LMX		Employee-rated LMX		Employee autonomy		Employee competence		Employee relatedness	
	*b*	*SE*	*b*	*SE*	*b*	*SE*	*b*	*SE*	*b*	*SE*
(Intercept)	3.52***	0.47	4.16***	0.49	2.44***	0.46	2.16***	0.41	3.93***	0.49
Leader gender	0.03	0.11	0.11	0.11	−0.16^†^	0.09	0.02	0.09	0.13	0.09
Employee gender	0.03	0.12	–0.10	0.13	0.23^†^	0.12	–0.03	0.08	0.06	0.11
Leader tenure	−0.01^†^	0.01	−0.01^†^	0.01	–0.00	0.00	–0.01	0.00	0.00	0.01
Employee tenure	–0.00	0.00	0.01	0.00	–0.00	0.00	0.00	0.00	0.00	0.00
SPOC	0.00	0.00	–0.01	0.00	–0.00	0.00	0.00	0.00	−0.01*	0.00
Leader autonomy	0.04	0.06	−0.11^†^	0.07	0.02	0.06	−0.23***	0.06	0.02	0.06
Leader competence	0.13	0.08	0.24**	0.09	0.05	0.07	0.88***	0.06	0.05	0.07
Leader relatedness	0.16*	0.06	0.14*	0.07	0.03	0.06	–0.07	0.06	–0.02	0.06
Leader-rated LMX					–0.07	0.04	–0.04	0.03	–0.09	0.04
Employee-rated LMX				0.42***	0.04	0.15***	0.03	0.33***	0.04	
Pseudo *r*^2^ lv1	0.13		0.04		0.13		0.42		0.15	
Pseudo *r*^2^ lv2	0.27		0.35		0.19		0.19		0.12	
Total *r*^2^	0.18		0.11		0.19		0.41		0.14	

^†^p < 0.10; *p < 0.05;**p < 0.01; ***p < 0.001. Total r^2^ = pseudo r^2^ level 1 × [1 - ICC(1)] + pseudo r^2^ level 2 × ICC(1), therefore only the total r^2^ (not the pseudo r^2^s) can be compared across models.

**TABLE 6:** Monte Carlo mediation indirect effects per psychological need.

Model	*Indirect effect [CI]*	*Total effect [CI]*	Mediation
**Leader competence**			
*/Employee-rated LMX/Employee autonomy*	0.11** [0.04; 0.18]	0.16* [0.01; 0.31]	Full
*/Employee-rated LMX/Employee competence*	0.04** [0.01; 0.07]	0.92*** [0.79; 1.04]	Partial
*/Employee-rated LMX/Employee relatedness*	0.09** [0.03; 0.15]	0.14 [−0.01; 0.29]	Full
**Leader relatedness**			
*/Employee-rated LMX/Employee autonomy*	0.06* [0.01; 0.12]	0.08 [−0.03; 0.21]	Full
*/Employee-rated LMX/Employee competence*	0.02* [0.01; 0.04]	−0.04 [−0.16; 0.07]	Full
*/Employee-rated LMX/Employee relatedness*	0.05* [0.01; 0.09]	0.03 [−0.08; 0.15]	Full

*p < 0.05; **p < 0.01; ***p < 0.001.

#### Direct Effects

Leader autonomy was only related to one employee psychological need, i.e., employee competence. Contrary to expectations, leader autonomy had a negative relationship with employee competence (*b* = −0.23, *p* < 0.001). Leader competence was positively related to employee competence (*b* = 0.88, *p* < 0.001), as well as to employee-rated LMX (*b* = 0.24, *p* < 0.05). Leader relatedness was positively associated with leader-rated LMX (*b* = 0.16, *p* < 0.05) and employee-rated LMX (*b* = 0.14, *p* < 0.05). Leader-rated LMX was not associated with employee autonomy, competence, or relatedness. Employee-rated LMX, however, had significant influences on all three psychological need dimensions: employee autonomy (*b* = 0.42, *p* < 0.05), employee competence (*b* = 0.15, *p* < 0.05), and employee relatedness (*b* = 0.33, *p* < 0.05).

Since leader autonomy was unrelated to both employee- and leader-rated LMX, only the indirect effects of leader competence and leader relatedness were calculated.

##### Leader Competence

Monte Carlo simulations showed that the indirect effect of leader competence on the employee autonomy was 0.11 (*p* < 0.05), 95% *CI* [0.04; 0.18]. The indirect effect on employee competence was 0.04 (*p* < 0.01), 95% *CI* [0.01; 0.07] and the indirect effect on employee relatedness was 0.09 (*p* < 0.001), 95% *CI* [0.03; 0.14]. See Tables 5, 6 and Figure 3 for the full results.

##### Leader Relatedness

The indirect effect of leader relatedness on employee autonomy was 0.06 (*p* < 0.05), 95% *CI* [0.01; 0.12]. The indirect effect on employee competence was 0.02 (*p* < 0.05), 95% *CI* [0.01; 0.05] and the indirect effect on employee relatedness was 0.05 (*p* < 0.01), 95% *CI* [0.01; 0.10]. See Tables 5, 6 and Figure 3 for the full results.

## Discussion

This paper examined the trickle-down of leader psychological need satisfaction *via* the dyadic process of LMX. Previous research devoted attention to how “positive” leadership styles and leader behaviors contribute to employee motivation and performance (Antonakis and Day, 2017), while less attention has been devoted to how a leader’s mindset influences employees (Sauer and Kohls, 2011) or trickles down the organization (Frazier and Tupper, 2018). Since leadership is an inherently relational social influence process (Uhl-Bien, 2006), it is relevant to study how exchanges with employees unfold (Cropanzano et al., 2017) from a dyadic (Krasikova and LeBreton, 2012; Gooty and Yammarino, 2011; Gooty et al., 2012; Tse et al., 2018) and multilevel perspective (Schreurs et al., 2014; Batistič et al., 2017). Our study was consistent with such perspectives and also answered calls in the trickle-down field for more work on the mediating mechanisms in the trickle-down process (Wo et al., 2019) and the integration of SDT and LMX theory (Andersen et al., 2020).

### Theoretical Implications

Consistent with SDT, our results show that the leader psychological need satisfaction predicted the employee psychological need satisfaction. This is in line with studies like Paas et al. (2020) that have observed a positive relationship between psychological need satisfaction and positive leadership behavior. In turn, the leader psychological need satisfaction also influenced both leader-rated LMX and employee-rated LMX. In other words, when leaders feel their psychological needs are satisfied, they are motivated in the well-being and growth of their employees in such a way that the overall LMX-quality, both leader and employee perceptions of LMX prosper. In line with the leadership perspective on psychological need satisfaction (e.g., Breevaart et al., 2014b; Chiniara and Bentein, 2016; Decuypere and Schaufeli, 2020, 2021), LMX also predicted the employee outcomes, i.e., employee psychological need satisfaction. However, this was only true for employee-rated LMX. Since psychological need satisfaction, like perceptions of LMX-quality, can be seen as “private” events, best judged by self-report questionnaires (Conway and Lance, 2010; Decuypere et al., 2018), we could have expected that employee perceptions of LMX-quality are better associated with (self-rated) employee outcomes than with leader perceptions. Likewise, only employee-rated LMX mediated the trickle-down effect of leader psychological need satisfaction on the employee psychological need satisfaction. In addition, the control variables did indicate a small association between leader tenure and LMX rated by both parties. A higher SPOC, which limits the chances of developing employee relationships, corresponded with a lower employee-rated LMX. This was in line with previous observations and theorizing (Graen and Scandura, 1987; Maslyn and Uhl-Bien, 2001; Cogliser et al., 2009; Sin et al., 2009; Schyns and Day, 2010). These findings lend support to our hypotheses concerning both a direct trickle-down effect of leader psychological need satisfaction, as well as an indirect effect through employee-rated LMX. Therefore, this study answered to calls on taking into account the underdeveloped leader perspective on LMX (Schyns and Day, 2010). Moreover, by taking a dyadic approach, we assured multiple perspectives on the leader–employee professional relationship were incorporated (Krasikova and LeBreton, 2012; Uhl-Bien, 2006; Schyns and Day, 2010; McCusker et al., 2019). Our results indicate that employee LMX-perceptions could be more influential for employee psychological need satisfaction.

Furthermore, we contribute to a more fine-grained understanding of the trickle-down effect of separate psychological needs by demonstrating some interesting patterns in our additional analysis of the separate need factors. First, leader autonomy need satisfaction was not related to the leader-rated LMX and only marginally (and negatively) associated with employee-rated LMX. Most notably, leader autonomy was associated with only one employee psychological need: we found a negative relationship with employee competence. This negative cross-domain influence is an unexpected finding. Perhaps a higher perceived level of freedom and decision-making latitude experienced by the leader is intimidating for an employee, hampering employee confidence, and thus lowering employee competence need satisfaction. Alternatively, perhaps leaders in our research context who feel like they have more decision-making freedom are somehow less inclined to support and help their employees. Following this line of thought, leaders who score high in autonomy need satisfaction may be characterized more by a laissez-faire leadership style, which is actually quite destructive for employees (Skogstad et al., 2007), and may hamper employee development (and thus competence need satisfaction).

Second, leader competence was both directly and indirectly related to the employee’ psychological needs: it was positively related to both employee competencies, as well as to the employee-rated LMX. This indicates that leaders who feel competent can increase the employees’ subjective feelings of competence. Perhaps leaders accomplish this by actually providing support (e.g., training) to their employees; or perhaps leaders who feel competent (and confident) themselves are simply able to instill or inspire the same feeling of competence (and confidence in abilities) in their employees. Leader competence need satisfaction is also the only psychological need that trickles down directly to employees. In addition, through increasing the employee-rated LMX, leader competence need satisfaction also influences all three employee needs indirectly. Again, this shows that a leader who feels competent impacts employee need satisfaction through enhanced employee relationships.

Third, leader relatedness was positively associated with the leader-rated LMX and employee-rated LMX. Indirectly, through its association with employee-rated LMX, leader relatedness impacts all three employee needs, indicating the importance of how connected a leader feels with employees for employee outcomes. In sum, these additional analyses revealed an important and rather unexpected research finding that contributes to the literature and evokes further questions: separate psychological needs do not necessarily follow the same trickle-down path. Leaders’ autonomy only has a direct influence, while leader competence both directly and indirectly influences on the employees’ psychological need satisfaction. Leader relatedness, then, only indirectly influences on the employee needs. Furthermore, the additional analyses mostly indicate the importance of leader competence need satisfaction for employee psychological need satisfaction. A leader who feels competent will enhance employee relatedness, autonomy, and competence. Previous research indeed indicated all kinds of negative employee effects in the absence of leader competence. For example, a lack of self-perceived leader competence is related to leader aggression (Fast and Chen, 2006). It will also enhance the leaders’ stress, which has demonstrable effects on employee stress and affective well-being (Skakon et al., 2010).

### Limitations and Future Research

There are also some limitations to our research design that provide opportunities for future research. First, despite our multilevel and multisource data, our design was cross-sectional. Therefore, the hypothesized associations were based solely on theoretical deliberations. In addition, we cannot make actual causal inferences based on our results. Specifically, this also means that a trickle-up effect based on our model cannot be excluded. Relatedly, LMX might take time to develop, and even though we controlled for tenure at both levels, our cross-sectional design does not take evolution over time into account (Lord, 2019; McCusker et al., 2019). Regrettably, our design does not permit us to investigate the complex interplay of our focal variables in a dynamic way. Daily differences in how leaders feel may influence daily leader need satisfaction and trickle-down to daily employee need satisfaction. Or daily differences in employees’ need satisfaction may influence the leaders’ need satisfaction *via* LMX perceptions, particularly if both parties had a lot of contacts. Diary studies or experience sampling could be an interesting future research avenue in order to explore these effects (see e.g., Tims et al., 2011; Breevaart et al., 2014b; De Gieter et al., 2017; Hetland et al., 2018), specifically with regards to fluctuations in leaders’ psychological need satisfaction and the effects on employees, as well as trickle-up influences. In addition, future research could account for the effect of cultural differences in the perceptions of LMX-agreement on certain aspects of leader behavior (Karakitapoğlu-Aygün et al., 2021).

Second, our results indicate that there is a differential strength of effects with regards to leader-rated variables and employee-rated variables. Although this is to be expected, i.e., leader-perceptions will be more important for leader outcomes and vice versa, common rater bias cannot be entirely excluded.

Third, our specific research context, i.e., an elderly care home in Flanders with a predominantly female staff, is a highly stressful environment (Smith, 2014; Tahghighi et al., 2017; Chênevert et al., 2019). It is also a quite specific environment and results may not be generalizable. Future research could aim at replicating our research results in different contexts.

Fourth, a leader–employee relationship does not form within a vacuum; the larger team context matters a lot as well (see e.g., Liang et al., 2021). One can argue that a leader may also influence positive emotions or more general mood in the work team; and the team members will also influence each other. Research has, for example, shown that supportive leadership fosters team-member exchange (TMX) (Kim et al., 2021); for example, through supporting a general collegial atmosphere, or through providing all the resources that teams need. In addition, we would expect that TMX may be a more proximal influence than LMX in contexts where leaders have a higher SPOC and employees have more contact with team members. Relatedly, TMX may also be more important in contexts where emotional labor is necessary as well (Kang and Jang, 2022). Future research may also want to take this into account.

Last, we also urge future researchers to dive into the complex inter-relationships between different psychological needs, especially in light of the unexpected results. Reverse causality or “trickle-up” effects are also an interesting research avenue (Wo et al., 2019) that can take into account the dyadic effect of LMX; and therefore, the effect of employees can have on leaders (Uhl-Bien, 2006).

### Practical Implications

From a practical point of view, our results indicate that leader’s psychological need satisfaction matters for leaders as well as employees. Therefore, both leaders and their organizations have a responsibility in supporting psychological need satisfaction. Our results indicate that organizations should not only develop practices and policies directly targeted to help those at the lowest level in the organization succeed, but also for their leaders, as this will naturally trickle down in the organization.

Overall, organizations should focus on increasing leaders’ competence (e.g., through more education or mentoring programs) and relatedness (e.g., through more informal gatherings or activities) since this will enhance well-being and performance for leaders themselves (Deci and Ryan, 2000), as well as trickle down to their employees. Specifically, we found that leader competence was the only need that trickled down directly to employees – indicating that leaders’ sense of effectivity at the work floor is of paramount importance and should be higher on organizations’ agendas. Based on our findings, we would also warn organizations for the potential negative effects of leader autonomy on employee competence. Even when leaders are granted autonomy that foster the fulfillment of the autonomy need, leaders should still make sure that employees feel competent and have access to all the training and help, they may need. Furthermore, leaders may want to focus on improving the relationship quality with their employees, e.g., through increasing the opportunities for (informal) positive exchanges.

In addition, our study also indicates the importance of leaders’ self-care and self-monitoring with regards to their psychological needs: are they still feeling connected (i.e., relatedness need satisfaction)? Is there something they feel insecure about and could address (i.e., competence need satisfaction)? Management books have long advocated for leader self-development *and* self-care in order to be successful (Latham, 2018). Our research indicates that this trickles down and is also important for employees’ perceptions of the LMX quality, their psychological need satisfaction, and, consequently, their success.

## Conclusion

Despite psychological need satisfaction’s mounting prominence in leadership literature, it has mostly been examined using a single-level approach and as a consequence of leader behavior. The present study has strived to address these shortcomings by looking at how psychological needs satisfaction transfers from leaders to employees through the influence of LMX processes. In a study of Belgium nurse-leader dyads, the results confirmed that psychological need satisfaction trickles down through how employees rate their relationship with their leaders (LMX). Further inspection demonstrated the discrepant influences for the different psychological needs. While leader competence directly influenced the employees’ need satisfaction, leader relatedness only did so mediated by LMX. Overall, our research contributes both to theoretical developments integrating need satisfaction within the trickle-down paradigm, while also offering suggestions for practice. From a theoretical point of view, this study confirms that (1) psychological need satisfaction might be an important element in determining how leaders act and employees react to leaders, as well as (2) that LMX constitutes an important mechanism underlying this phenomenon. From a practical point of view, our results indicate that in their design of organizational practices and policies organizations should not only target employees, but also leaders’ competence and relatedness, since such investments are likely to transmit to employees through a trickle-down process.

## Data Availability Statement

The raw data supporting the conclusions of this article can be obtained from the first author.

## Ethics Statement

Ethical review and approval was not required for the study on human participants in accordance with the local legislation and institutional requirements. The patients/participants provided their written informed consent to participate in this study.

## Author Contributions

AD was responsible for the research question, data-collection, data-analysis, and for writing the manuscript. RB was responsible for data-collection, initial data-analysis, and for writing and reviewing parts of the manuscript. MA was responsible for overseeing the data-collection and for final approval of the manuscript. All authors contributed to the article and approved the submitted version.

## Conflict of Interest

The authors declare that the research was conducted in the absence of any commercial or financial relationships that could be construed as a potential conflict of interest.

## Publisher’s Note

All claims expressed in this article are solely those of the authors and do not necessarily represent those of their affiliated organizations, or those of the publisher, the editors and the reviewers. Any product that may be evaluated in this article, or claim that may be made by its manufacturer, is not guaranteed or endorsed by the publisher.

## Footnotes

1 These authors adapted the original seven-item scale from Liden et al. (1993). They split one item (“Do you usually feel that you know where you stand. Do you usually know how satisfied your immediate supervisor is with what you do?”) into two separate items: (1) “I usually know where I stand with my manager” and(2) “I usually know how satisfied my manager is with me” (see Bauer and Green, 1996, p. 1554).
